# SIK inhibitor HG-9-91-01 suppresses the pathogenic activity of fibroblast-like synoviocytes in rheumatoid arthritis

**DOI:** 10.1136/rmdopen-2026-007000

**Published:** 2026-08-20

**Authors:** Xiaohao Wang, Yuanyuan Li, Hanchao Li, Lingfei Mo, Xinyi Liu, Zechao Qu, Jing Wang, Liang Yan

**Affiliations:** 1 Department of Spine Surgery, Honghui Hospital of Xi’an Jiaotong University, Xi’an, Shaanxi, China; 2 Department of Rheumatology and Immunology, First Affiliated Hospital of Xi’an JiaoTong University, Xi’an, Shaanxi, China

**Keywords:** Rheumatoid Arthritis, Fibroblasts, Arthritis

## Abstract

**Objectives:**

Rheumatoid arthritis (RA) is a chronic autoimmune disorder characterised by synovial inflammation and joint destruction, in which fibroblast-like synoviocytes (FLS) play a pivotal role through their hyperproliferative, invasive and inflammatory properties. The salt-inducible kinase (SIK) family regulates inflammatory responses, yet the role of its isoform SIK3 in RA and the therapeutic potential of its inhibition remain unclear. This study investigates the effects of HG-9-91-01, a potent SIK inhibitor, on RA-FLS pathogenicity and disease progression.

**Methods:**

The impact of HG-9-91-01 on human MH7A cells was assessed using cell counting kit-8, 5-ethynyl-2′-deoxyuridine, flow cytometry, wound healing and Transwell assays. RNA sequencing, bioinformatics analyses and western blot analysis were employed to explore the underlying mechanisms. The therapeutic efficacy of HG-9-91-01 was evaluated in a murine collagen-induced arthritis (CIA) model through clinical scoring, histopathology and micro-CT imaging.

**Results:**

SIK3 was significantly upregulated in RA synovial tissues and correlated with disease activity. HG-9-91-01 potently inhibited MH7A cell proliferation, migration and invasion while promoting apoptosis. Transcriptomic and molecular analyses revealed that these effects were mediated through the concurrent suppression of the phosphoinositide 3-kinase-protein kinase B (PI3K-Akt) and nuclear factor kappa B (NF-κB) signalling pathways, leading to downstream modulation of Bcl-2-associated X protein, B-cell lymphoma 2, inducible nitric oxide synthase, tumour necrosis factor-α and matrix metalloproteinase-9. In CIA mice, HG-9-91-01 administration markedly alleviated arthritis severity, synovitis, cartilage degradation and bone erosion.

**Conclusions:**

Our findings demonstrate that HG-9-91-01 attenuates RA progression by directly targeting FLS pathogenicity via dual inhibition of PI3K-Akt and NF-κB signalling, highlighting the therapeutic potential of SIK family inhibition in RA.

**WHAT IS ALREADY KNOWN ON THIS TOPIC**

- Rheumatoid arthritis fibroblast-like synoviocytes (RA-FLS) migration and invasion contribute directly to synovial pannus formation and joint destruction in RA.
- The salt-inducible kinase (SIK) family kinases regulate inflammatory signalling and immune responses, but their role in RA-FLS remains insufficiently defined.

**WHAT THIS STUDY ADDS**

- This study demonstrates that SIK3 is upregulated in RA synovium and that the SIK inhibitor HG-9-91-01 suppresses MH7A cell viability, proliferation, migration and invasion, promotes apoptosis and ameliorates clinical and structural damage in collagen-induced arthritis mice.
- These effects are associated with reduced phosphoinositide 3-kinase-protein kinase B/mechanistic target of rapamycin and nuclear factor kappa B pathway activation.

**HOW THIS STUDY MIGHT AFFECT RESEARCH, PRACTICE OR POLICY**

- The findings support further investigation of SIK family inhibition as a potential therapeutic direction for RA, while highlighting the need for validation in primary RA-FLS, immune-cell profiling and comprehensive safety assessment before clinical translation.

## Introduction

 Rheumatoid arthritis (RA) is a chronic autoimmune disease characterised by persistent synovial inflammation and progressive joint damage.^1 2^ RA fibroblast-like synoviocytes (RA-FLS) are central to its pathogenesis and have an invasive phenotype, characterised by enhanced migration and invasive behaviour.^3 4^ These abnormal cellular properties drive synovial hyperplasia and pannus formation, destroying cartilage and bone.^5^ Continuous migration and invasion of RA-FLS aggravate joint erosion and contribute to the chronicity of the disease,^6^ making these cellular processes potential therapeutic targets for controlling RA progression.

Current pharmacological management of RA primarily focuses on immunomodulation and inflammatory suppression. Conventional synthetic disease-modifying antirheumatic drugs (csDMARDs), such as methotrexate, effectively control symptoms in many patients, but a considerable proportion experience inadequate responses or loss of efficacy over time.^7 8^ Biological DMARDs (bDMARDs) targeting tumour necrosis factor (TNF)-α and interleukin (IL)-6 have transformed treatment paradigms, yet acquired resistance leads to suboptimal responses in approximately 40% of patients.^9 10^ Janus kinase (JAK) inhibitors have expanded therapeutic options, although their clinical application is limited by potential cardiovascular risks and increased susceptibility to opportunistic infections.^11 12^ Importantly, despite their distinct mechanisms, all these agents predominantly target immune activation pathways and show limited direct efficacy against the core pathogenic features of RA-FLS—particularly their persistent migratory and invasive capacities. This limitation may explain the disconnect between clinical improvement and continued radiographic progression in some patients, underscoring the urgent need for novel strategies that directly address synovial invasion and joint destruction.

Salt-inducible kinases (SIKs), a subfamily of the AMP-activated protein kinase family, have emerged as critical regulators of inflammatory signalling and cellular metabolism, with three isoforms (SIK1, SIK2 and SIK3) implicated in diverse biological processes including cell growth and immune responses.^13–17^ In the context of RA, pan-SIK inhibition has recently shown therapeutic promise, as the inhibitor YKL-05-099 alleviated joint destruction and synovial inflammation in CIA mice by promoting macrophage efferocytosis.^18^ These findings suggest that targeting SIK family kinases represents a potential anti-inflammatory and bone-protective strategy for RA. However, the specific role of SIK3, particularly its involvement in the pathogenic migration, invasion and inflammatory activation of RA-FLS, remains poorly defined.

HG-9-91-01 is a potent and selective small-molecule inhibitor of the SIK family, with accumulating evidence supporting its robust anti-inflammatory and immunomodulatory properties.^19 20^ In oncology research, this compound has demonstrated efficacy in suppressing tumour progression through the induction of apoptosis and inhibition of cancer cell migration and invasion.^21^ In inflammatory context, its therapeutic benefits are primarily mediated through modulation of key signalling pathways and suppression of pro-inflammatory cytokine production.^22 23^ Despite these advances, the specific impact of HG-9-91-01 on the migratory and invasive behaviours of RA-FLS—critical effector cells in RA joint destruction—and the precise molecular mechanisms involved remain incompletely characterised and merit further systematic investigation.

Based on this rationale, we hypothesised that pharmacological inhibition of SIKs by HG-9-91-01 could directly target the aggressive phenotype of RA-FLS and ameliorate RA progression. In this study, we first identified SIK3 as a disease-associated isoform upregulated in RA synovium. Using the MH7A human RA-FLS cell line and a murine collagen-induced arthritis (CIA) model, we systematically investigated the therapeutic effects of HG-9-91-01. Our results demonstrate that HG-9-91-01 effectively suppresses RA-FLS proliferation, migration and invasion while promoting apoptosis. Mechanistically, these effects are mediated through the concurrent inhibition of the phosphoinositide 3-kinase-protein kinase B (PI3K-Akt) and nuclear factor kappa B (NF-κB) signalling pathways. Our findings nominate SIK inhibition as a novel and promising therapeutic strategy for RA.

## Materials and methods

### Antibodies and reagents

HG-9-91-01 (purity ≥99.77%, Chemical Abstracts Service (CAS) no. 1456858-58-4, molecular weight 567.68 Da) was purchased from MedChemExpress (MCE, Monmouth Junction, New Jersey, USA). A 1 mmol/L stock solution was prepared by dissolving the compound in dimethyl sulfoxide (analytical grade, ≥99.9%) and stored at −80°C in the dark. Basic culture media, fetal bovine serum (FBS) and penicillin-streptomycin (PS) solution were obtained from Gibco (Thermo Fisher Scientific, Waltham, Massachusetts, USA). The cell counting kit-8 (CCK-8) was purchased from TargetMol (Boston, Massachusetts, USA). The fluorescein isothiocyanate (FITC)-labelled annexin V apoptosis detection kit I was sourced from BD Biosciences (San Jose, California, USA). Transwell chambers were acquired from Corning (Corning, New York, USA). Primary antibodies against PI3K, Akt, mechanistic target of rapamycin (mTOR), NF-κB and β-actin, as well as goat antimouse and goat antirabbit secondary antibodies, were all purchased from Chengdu Zhengneng Biotechnology (Chengdu, Sichuan, China). Bovine type II collagen (CII), complete Freund’s adjuvant (CFA) and incomplete Freund’s adjuvant (IFA) were obtained from Chondrex (Redmond, Washington, USA).

### Clinical samples

Human synovial tissue samples were collected from patients with RA who were diagnosed in accordance with the 2010 American College of Rheumatology/European League Against Rheumatism (EULAR) classification criteria and underwent total joint replacement surgery at First Affiliated Hospital of Xi’an Jiaotong University, as well as age-matched and gender-matched healthy controls who underwent knee arthroscopy for traumatic meniscal injury (without a history of autoimmune diseases, inflammatory disorders or joint pathology). Immediately after surgical resection, synovial tissue specimens were rinsed with sterile phosphate-buffered saline (PBS; pH 7.4) to remove residual blood and debris: a portion was snap-frozen in liquid nitrogen and stored at −80°C for subsequent molecular biology experiments, while another portion was fixed in 4% paraformaldehyde at 4°C for 24 hours for histological analysis. Detailed clinical data of all participants were systematically recorded.

### Cell culture

The human RA-FLS cell line MH7A was purchased from the American Type Culture Collection (Manassas, Virginia, USA). For cell passage, cells were rinsed three times with sterile PBS and digested with 0.25% trypsin-EDTA solution at 37°C for 3 min. The digestion was terminated by adding an equal volume of Dulbecco’s modified Eagle medium (DMEM) supplemented with 10% FBS. Subsequently, cells were cultured in complete DMEM medium (containing 10% FBS and 1% PS) in a humidified incubator maintained at 37°C with 5% CO_2_.

### The relative messenger RNA expression levels of target genes were determined by reverse transcription quantitative PCR

Total RNA was extracted from synovial tissues of patients with RA and healthy controls (HCs), followed by reverse transcription into complementary DNA. Quantitative PCR was performed using SYBR Green Master Mix on a Bio-Rad PCR system. Gene-specific primer sequences are listed in table 1. Relative gene expression levels were normalised to glyceraldehyde-3-phosphate dehydrogenase and calculated using the 2−ΔΔCT method. Each sample was detected with three technical replicates and the entire experiment was conducted in three independent biological replicates.

**Table 1:** Primer sequences for reverse transcription quantitative PCR analysis

Gene	Forward primer	Reverse primer
SIK1	TGGACGTCTGGAGCCTCGGT	AGAGTGGGGTCGGCCTGCAT
SIK2	TGAGCAGGTTCTTCGACTGAT	AGATCGCATCAGTCTCACGTT
SIK3	TCCCCACTTGTCACCATGAC	GAGCGATGCTGGTCAGGTAC
GAPDH	ATGACCCCTTCATTGACCTCA	GAGATGATGACCCTTTTGGCT

GAPDH, glyceraldehyde-3-phosphate dehydrogenase; SIK, salt-inducible kinase.

### Cell viability and proliferation assays

The effects of HG-9-91-01 on cell viability and proliferation were assessed. For viability, MH7A cells seeded in 96-well plates were treated with the compound (0.5–4 µM) for 24 hours. After treatment, cell viability was measured using the CCK-8 according to the manufacturer’s protocol and the absorbance was read at 450 nm. For proliferation, cells cultured in 24-well plates were treated similarly, followed by incubation with 10 µM 5-ethynyl-2′-deoxyuridine (EdU) for 2 hours. Cells were then fixed, stained using the EdU imaging kit and counterstained with Hoechst 33 342. EdU-positive cells were imaged under a fluorescence microscope (Olympus BX63) and quantified to calculate the proliferation rate. Each experimental group contained three replicate wells per plate and the cell viability assay was repeated in three independent biological experiments.

### Flow cytometry apoptosis analysis

Following the protocol of the Apoptosis Detection Kit (BD Biosciences), MH7A cells were preprocessed as above, and washed, resuspended, stained with 5 μL annexin V/FITC for 10 min and then added 5 μL propidium iodide. The number of apoptotic cells was measured using a flow cytometer (NovoCyte Advanteon Flow Cytometer). Each group was set up with three parallel samples, and the apoptosis assay was performed in three independent biological replicates.

### Wound healing assay

MH7A cells were seeded in 6-well culture plates and incubated until a confluent monolayer was formed. To eliminate the confounding effect of cell proliferation on wound closure, cells were pre-treated with 10 μg/mL mitomycin C (Sigma-Aldrich) at 37°C for 2 hours. Following mitomycin C treatment, the cell monolayer was gently rinsed twice with PBS to completely remove residual mitomycin C. A uniform straight scratch was generated across the cell monolayer using a sterile 200 μL pipette tip. Detached floating cells were removed by gentle PBS washing, and fresh serum-free medium supplemented with the corresponding concentrations of HG-9-91-01 (0, 0.5, 1, 2 and 4 μM) was subsequently added to each well. The cells were then cultured at 37°C in a humidified incubator with 5% CO_2_. Microscopic images of the scratched areas were captured immediately after scratching (0 hour) and at 24 hours postinjury using an inverted microscope. The wound closure area was quantitatively analysed using ImageJ. All experimental procedures were performed in triplicate with three independent biological replicates.

### Transwell invasion assay

To exclude the confounding effect of cell proliferation on invasive capacity, MH7A cells were first pretreated with 10 μg/mL mitomycin C (Sigma-Aldrich) in complete medium at 37°C for 2 hours. Subsequently, the treated cells were trypsinised and resuspended in serum-free DMEM to prepare single-cell suspensions, and the cell density was adjusted to 1×10⁵ cells/mL for subsequent assays. Matrigel (BD Biosciences) was diluted with ice-cold serum-free DMEM at a ratio of 1:8, and 40 μL of the diluted Matrigel solution was added to the upper chamber of 8 μm-pore Transwell inserts. The inserts were incubated at 37°C for 4–6 hours to allow full Matrigel solidification. For chemotaxis induction, 500 μL of DMEM supplemented with 10% FBS was added to the lower chamber of the Transwell plate. Thereafter, 200 μL of cell suspension containing the corresponding concentrations of HG-9-91-01 (0, 0.5, 1, 2 and 4 μM) was seeded into each Matrigel-precoated upper chamber. The plates were incubated at 37°C in a humidified atmosphere with 5% CO_2_ for 24 hours. After incubation, non-invasive cells on the upper membrane surface were carefully wiped off with a cotton swab. The invasive cells on the lower membrane surface were fixed with methanol for 30 min and stained with 0.1% crystal violet solution at room temperature for 15 min. Microscopic images were captured using an inverted microscope, with at least five random fields collected per insert. The number of invasive cells was quantified using ImageJ. All experimental procedures were performed in triplicate with three independent biological replicates.

### Western blot analysis

MH7A cells in 6-well plates were incubated with different concentrations of HG-9-91-01 for 24 hours. Cells were lysed using radioimmunoprecipitation assay lysis buffer supplemented with phenylmethylsulfonyl fluoride and phosphatase inhibitors, following the manufacturer’s recommended protocol. Protein concentrations were quantified using a BCA assay kit (Thermo Fisher Scientific). After protein sampling, the proteins were electrophoresed at 90 V for 20 min and then transferred to 190 V for 50 min. Next, they were transferred to polyvinylidene fluoride membranes at 400 mA for 40 min and enclosed in a sealing solution for 15 min. After that, they were incubated with the primary antibody at 4°C overnight. Tris-buffered saline with Tween 20 was rinsed three times, followed by incubation with the secondary antibody at room temperature for 50 min. Enhanced chemiluminescence solution was used for exposure on a gel imaging analysis system. The grey value of each band was analysed using ImageJ.

### Bioinformatics and molecular docking analyses

RNA sequencing (RNA-seq) data from control and HG-9-91-01-treated MH7A cells were analysed to identify differentially expressed genes (DEGs) using the DESeq2 R package, with significance thresholds set at adjusted p value (padj) padj <0.05 and log2 fold change (|log2FC|) >1.5. Functional enrichment of DEGs was performed via Gene Ontology (GO) and Kyoto Encyclopedia of Genes and Genomes (KEGG) pathway analyses using the clusterProfiler package, and coordinated pathway changes were further assessed by Gene Set Enrichment Analysis (GSEA) against the MSigDB Hallmark gene set. To predict the interaction between HG-9-91-01 and SIK3, molecular docking was conducted using AutoDock Vina after preparing the ligand (from PubChem) and protein (from UniProt) structures, with a binding energy < −5 kcal/mol indicating a stable interaction.

### Single-cell RNA sequencing analysis

To evaluate the expression patterns of SIK family members in RA synovial tissue, publicly available single-cell RNA-seq datasets were retrieved from the Gene Expression Omnibus. Synovial samples from patients with RA were obtained from GSE200815, whereas healthy synovial samples were obtained from GSE246416. Raw sequencing data (FASTQ files) were downloaded from the Sequence Read Archive and processed using a unified analytical pipeline.

Raw sequencing reads were aligned to the human reference genome (GRCh38) and gene expression matrices were generated using Cell Ranger (10x Genomics) with default parameters. Individual samples passing quality control were imported into Seurat (V.5.0) in R. Cells with low-quality transcriptomes, excessive mitochondrial gene expression or extremely low/high gene counts were excluded prior to downstream analyses. Gene expression values were log-normalised, and the top 2000 highly variable genes were identified for dimensionality reduction.

To minimise technical differences between datasets generated from independent studies, batch effects were corrected using the Harmony algorithm based on sample identity. Principal component analysis was performed using the corrected embeddings, followed by Uniform Manifold Approximation and Projection for visualisation. Cell clusters were identified using the Louvain algorithm and annotated according to canonical marker genes reported in previous studies.

### Induction of CIA mice

Healthy male DBA/1 mice were purchased from GemPharmatech (Nanjing, Jiangsu, China) and housed in a specific pathogen-free animal facility at Xi’an Honghui Hospital under standardised conditions (temperature: 22±2°C, relative humidity: 50%±10%, 12 hours light/12 hours dark cycle) with ad libitum access to sterile food and filtered water throughout the experiment. Animals were acclimatised to the housing conditions for 1 week before the initiation of the experiments. The CIA model was established via two-step immunisation. For the first immunisation (day 0), CFA and CII (1:1) were fused and 100 μL was injected subcutaneously 2 cm from the root of the tail of the mice. Subsequently, for booster immunisation on day 21, CII was emulsified with IFA at a 1:1 ratio. After shaving the dorsal and caudal root regions of the mice, a total volume of 100 μL of the CII-IFA emulsion was administered via subcutaneous injection at these sites.

After the onset of arthritis symptoms, mice were randomly allocated into three groups (n=6 per group): normal group, CIA model group and HG-9-91-01 treatment group. The sample size was selected based on previous studies using the CIA model and our previous experimental experience. Mice in the normal and CIA model groups were administered with equal volumes of 0.9% sterile normal saline via tail vein injection, while mice in the HG-9-91-01 group received tail vein injection of HG-9-91-01 at a dosage of 10 mg/kg body weight once daily for 28 consecutive days. At the end of the experiment, all mice were anaesthetised by isoflurane inhalation, and synovial tissues isolated from affected joints were harvested for subsequent histological and molecular biological analyses. Animals were monitored daily throughout the experiment for general health status, body condition and signs of pain or distress. No animals or experimental data were excluded from the analysis, and no unexpected adverse events occurred during the study.

### Radiographic examination

Paw samples were harvested from euthanised mice and immediately fixed in 4% paraformaldehyde. Micro-CT analysis was performed using a SKYSCAN 1172 scanner (Bruker, Kontich, Belgium) with standardised scanning parameters (voltage 70 kV, current 114 μA, voxel size 18 μm) to ensure imaging consistency. After scanning, three-dimensional reconstructions of the paw bones were generated using the manufacturer’s software. Quantitative bone parameters were calculated to assess bone integrity, including bone volume/tissue volume (BV/TV) and trabecular thickness (Tb.Th).

### Measurement of arthritis score and paw swelling

The arthritis score was evaluated every 3 days. Two experienced researchers blinded to group allocation independently assessed arthritis severity in mice using a validated visual scoring system (0–4 points) based on joint erythema and swelling, with specific criteria defined as follows: 0=no erythema or swelling in any paw joint; 1=mild swelling limited to the interphalangeal joints of the hind paws; 2=moderate swelling and erythema involving both the interphalangeal joints and plantar region of the hind paws; 3=severe swelling extending from the metatarsophalangeal joints to the ankle joint of the hind paws; 4=extensive erythema and severe swelling affecting all hind paw joints, including the ankle, with impaired movement.

### Histological examination

Mice were euthanised via overdose of isoflurane anaesthesia. The ankle joint tissues were harvested, fixed in 4% paraformaldehyde for 24 hours and then subjected to decalcification with 10% EDTA over 14 days. After decalcification, the hind limbs of the mice were first manipulated with paraffin for embedding, followed by sectioning and staining. H&E staining was performed to evaluate pathological changes in the ankle joint, while Safranin O-fast green staining was used to assess cartilage destruction.

### Statistical analysis

All quantitative data from in vitro cell experiments and in vivo animal experiments are presented as the mean±SD. All statistical analyses were performed using GraphPad Prism software (V.9.5.1). Specifically, the unpaired two-tailed Student’s t-test was used for statistical comparisons between two independent groups, while one-way analysis of variance followed by Tukey’s multiple comparisons post hoc test was applied for comparative analysis among three or more groups. For conventional phenotypic and functional experiments including cell viability, proliferation, apoptosis, migration, invasion, protein expression, animal behavioural and histological assessments, no additional false discovery rate (FDR) correction was performed; statistical significance was defined as a two-tailed p<0.05. For high-throughput RNA-seq differential expression analysis, the Benjamini-Hochberg (BH) FDR algorithm was strictly applied to correct multiple testing errors and eliminate false-positive results. Genes with padj <0.05 and absolute |log2FC| >1.5 were identified as significantly DEGs. Similarly, the BH-based FDR correction was also implemented for GO and KEGG functional enrichment analyses to adjust for multiple statistical testing, ensuring the reliability of enrichment results.

## Results

### SIK3 is upregulated in RA synovium and correlates with disease activity

**Figure 1:**
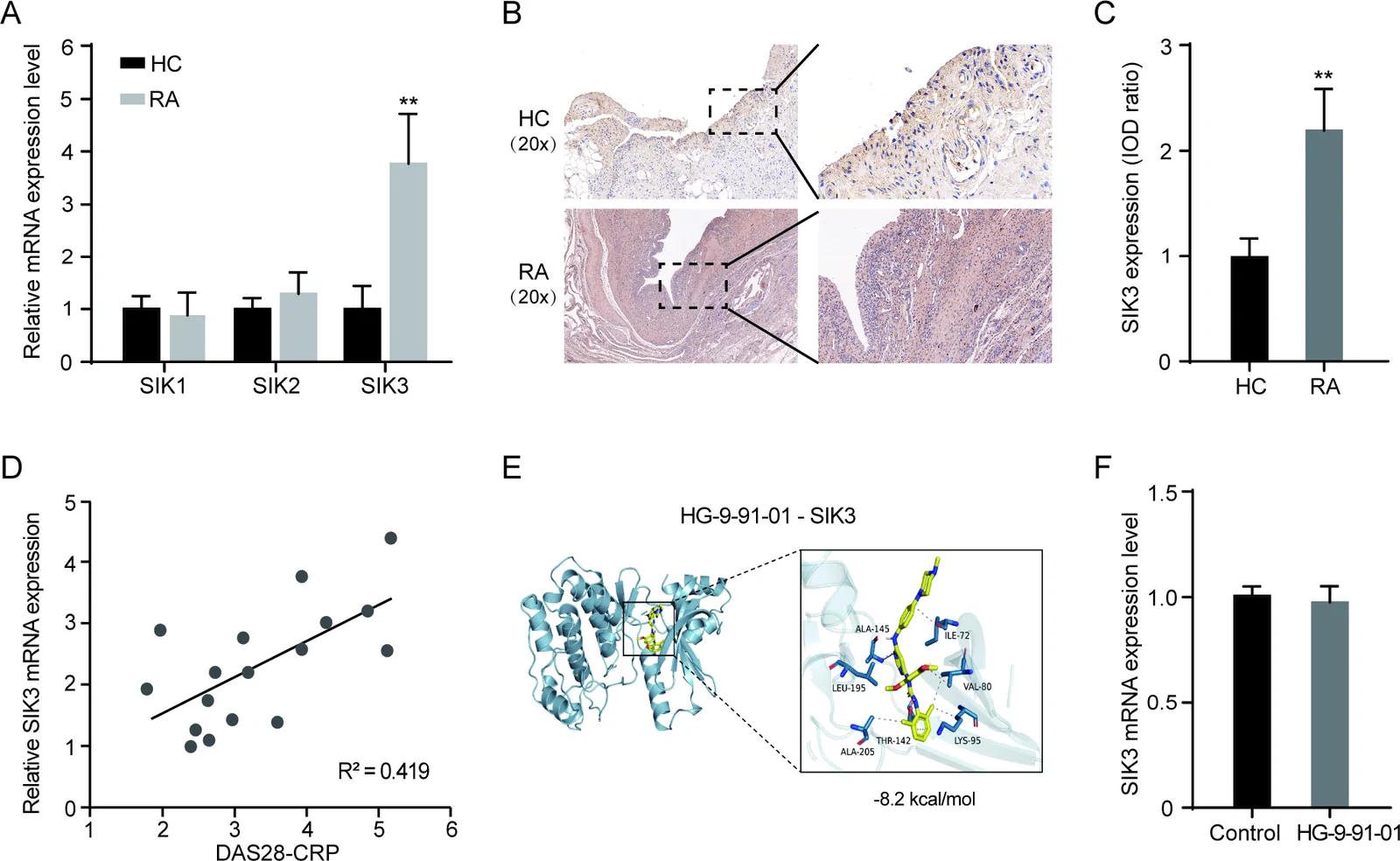
SIK3 is upregulated in RA synovium and correlates with disease activity. (**A**) Relative mRNA expression levels of SIK1, SIK2 and SIK3 in synovial tissues from HC and patients with RA. (**B**) Representative immunohistochemical staining of SIK3 protein in human synovial tissues. (**C**) Quantification of SIK3 IOD in synovial tissues from HC and patients with RA. (**D**) Correlation analysis between synovial SIK3 mRNA levels and clinical disease activity (DAS28-CRP) in patients with RA. (**E**) Molecular docking model of HG-9-91-01 binding to SIK3. (**F**) Effect of treatment with 1 μM HG-9-91-01 for 24 hours on SIK3 mRNA expression levels in MH7A cells. The above data are expressed as the mean±SD of three independent experiments; **p< 0.01 vs control group. DAS28-CRP, Disease Activity Score in 28 joints using C-reactive protein; HC, healthy controls; IOD, integrated optical density; mRNA, messenger RNA; RA, rheumatoid arthritis; SIK, salt-inducible kinase.

To elucidate the roles of SIK family members in RA, we first analysed the expression levels of SIK1, SIK2 and SIK3 in RA synovial tissues. The results showed that, unlike SIK1 and SIK2, SIK3 messenger RNA (mRNA) was significantly upregulated in RA synovial tissues (figure 1A), a finding further confirmed by immunohistochemical staining (figure 1B,C). Moreover, synovial SIK3 mRNA levels positively correlated with the Disease Activity Score in 28 joints using C reactive protein scores in patients with RA (figure 1D). These data suggest that SIK3 may be involved in synovial pathology in RA and is associated with disease activity. Then, we selected HG-9-91-01, an inhibitor of SIK family kinases, and molecular docking predicted a high-affinity interaction between them (binding energy: −8.2 kcal/mol; figure 1E). Notably, treatment with HG-9-91-01 did not alter SIK3 mRNA levels in MH7A cells (figure 1F), indicating that its effect on SIK3 is mediated through postfunctional level, rather than transcriptional control. In addition, analysis of a publicly available single-cell RNA-seq dataset revealed differential expression of SIK3 across immune-cell populations in RA synovium (online supplemental figure S1), suggesting that SIK3 may exert broader effects beyond synovial fibroblasts.

### HG-9-91-01 suppresses proliferation, migration, invasion and induces apoptosis of MH7A cells

**Figure 2:**
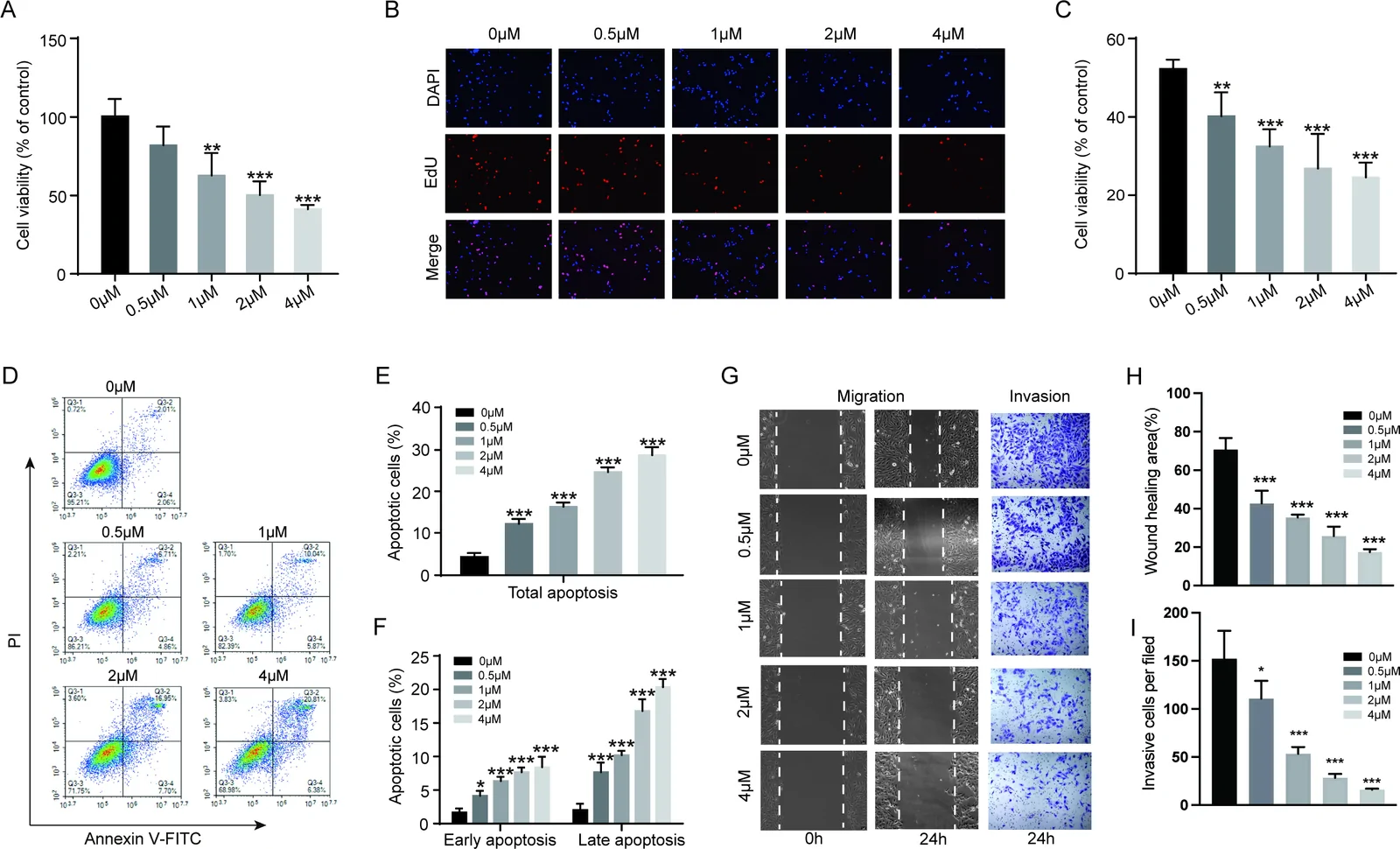
HG-9-91-01 inhibits proliferation, migration and invasion, and induces apoptosis in MH7A cells. (**A**) MH7A cell viability was analysed using the CCK-8 assay after HG-9-91-01 treatment. (**B–C**) Representative images and quantitative analysis of EdU staining (red) for proliferating MH7A cells. (**D–F**) MH7A cell apoptosis was measured using the annexin V-FITC/PI staining assay after HG-9-91-01 treatment. (**G–I**) Cell migration and invasion were assessed by wound healing and Matrigel-coated Transwell assays after HG-9-91-01 treatment. The above data are expressed as the mean±SD of three independent experiments; *p< 0.05, **p< 0.01, ***p< 0.001 vs control group. CCK-8, cell counting kit-8; EdU, 5-ethynyl-2′-deoxyuridine; FITC, fluorescein isothiocyanate; PI, propidium iodide.

To evaluate the antiproliferative effect of HG-9-91-01, MH7A cells were treated with the compound for 24 hours and cell viability was assessed using the CCK-8 assay (figure 2A) and the EdU assay (figure 2B,C). The results demonstrated that HG-9-91-01 reduced the viability of MH7A cells in a dose-dependent manner. We further examined whether HG-9-91-01 could induce apoptosis in MH7A cells. Flow cytometry analysis revealed an increase in the proportion of apoptotic cells on HG-9-91-01 treatment (figure 2D), with a dose-dependent rise in both early and late apoptotic populations (figure 2E,F). In vitro migration assays indicated that HG-9-91-01 markedly suppressed MH7A cell migration compared with the control group (figure 2G,H). Similarly, HG-9-91-01 significantly inhibited the invasion of MH7A relative to the control (figure 2G,I). Collectively, these findings suggest that HG-9-91-01 suppresses aggressive behaviours of MH7A cells in vitro.

### Transcriptome analysis reveals the anti-inflammatory mechanisms of HG-9-91-01 in MH7A cells

**Figure 3:**
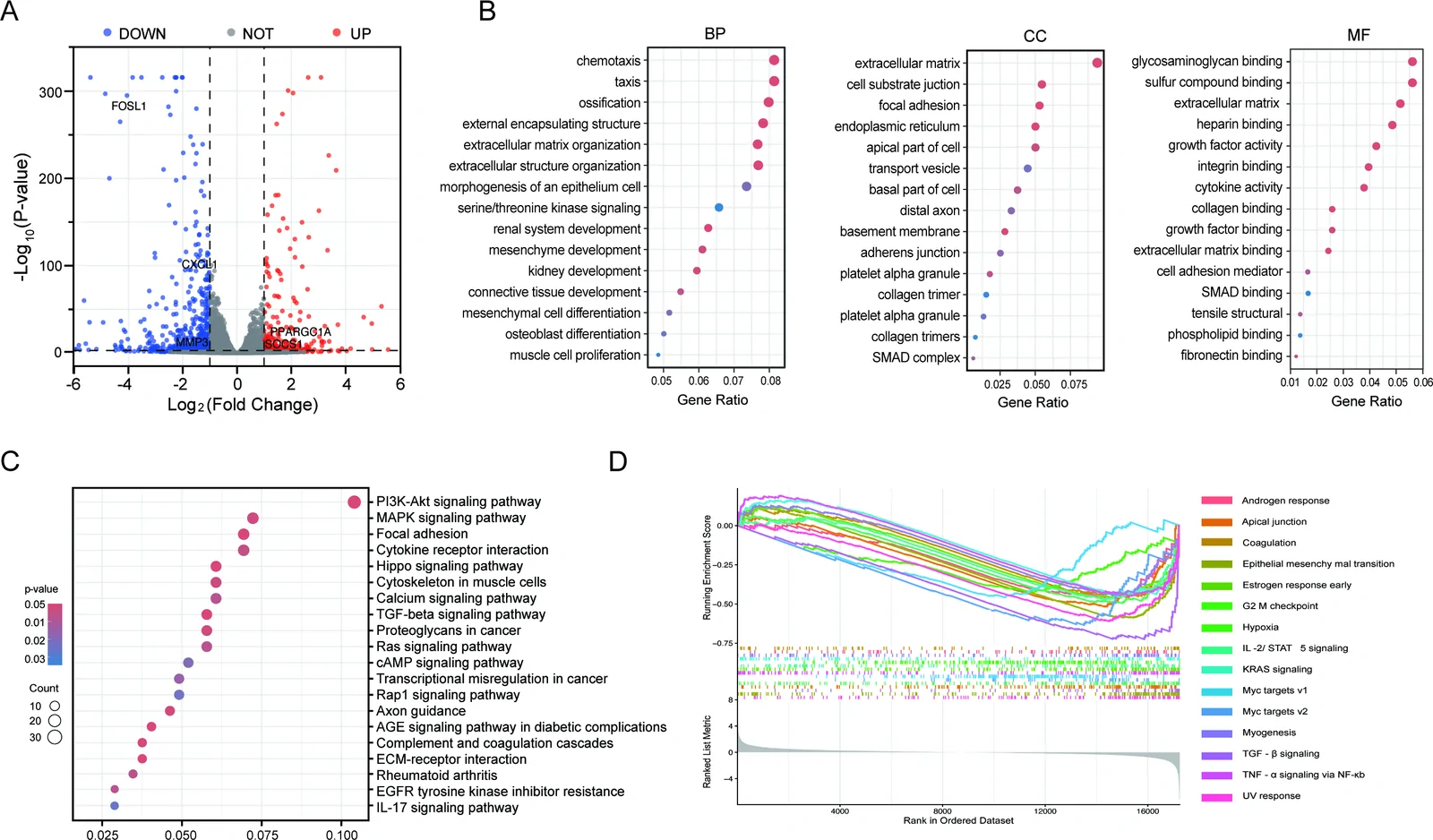
RNA-seq and functional enrichment in MH7A cells after HG-9-91-01 treatment. (**A**) Volcano map of DEGs in MH7A cells treated with HG-9-91-01. Blue represents downregulated genes and red represents upregulated genes. (**B**) GO functional enrichment analysis of DEGs. BP, CC and MF. (**C**) KEGG pathway enrichment analysis of DEGs. (**D**) GSEA pathway enrichment analysis of DEGs. BP, biological process; cAMP, cyclic AMP; CC, cellular component; DEG, differentially expressed gene; ECM, extracellular matrix; EGFR, epidermal growth factor receptor; GSEA, gene set enrichment analysis; GO, Gene Ontology; IL, interleukin; KEGG, Kyoto Encyclopedia of Genes and Genomes; MAPK, mitogen-activated protein kinase; MF, molecular function; NF-κB, nuclear factor kappa B; PI3K-Akt, phosphoinositide 3-kinase-protein kinase B; RNA-seq, RNA sequencing; SMAD, Sma- and Mad-related protein; TGF, transforming growth factor; TNF, tumour necrosis factor.

To elucidate the transcriptional mechanisms underlying the anti-inflammatory effects of HG-9-91-01, we performed RNA-seq on MH7A cells. This analysis identified 463 differentially expressed genes, revealing a coordinated shift in the transcriptional landscape. The expression of several pro-inflammatory genes, including CXCL1, FOSL1 and matrix metalloproteinase (MMP)3, was significantly suppressed, whereas inflammation-regulatory genes such as SOCS1 and PPARGC1A were markedly induced (figure 3A).

Functional enrichment analysis provided mechanistic insights into this response. GO analysis highlighted a significant enrichment of the DEGs in functions related to extracellular matrix organisation and constituent binding, cell adhesion structures and processes involving cell migration and differentiation (figure 3B). Importantly, KEGG pathway analysis highlighted significant enrichment in critical signalling pathways such as PI3K-Akt and MAPK (figure 3C). Furthermore, GSEA results confirmed the significant downregulation of the TGF-β, TNF-α and NF-κB signalling pathways (figure 3D). In summary, these transcriptomic data collectively suggest that HG-9-91-01 modulates a network of genes involved in extracellular matrix remodelling and key inflammatory signalling cascades.

### HG-9-91-01 inhibits PI3K-Akt and NF-κB signalling and modulates downstream effectors

**Figure 4:**
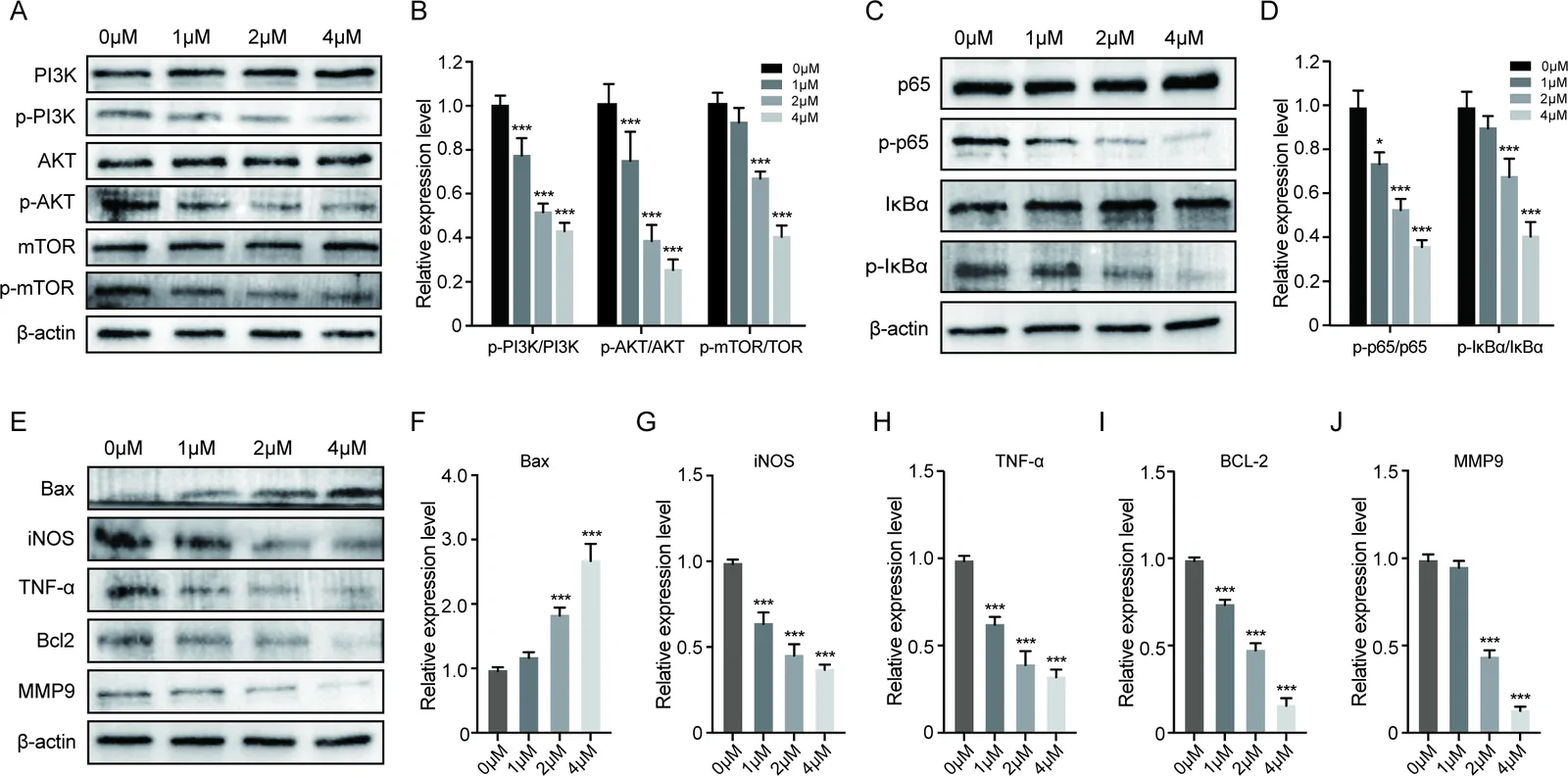
HG-9-91-01 inhibits the PI3K-Akt and NF-κB signalling pathways. (**A–B**) Western blot analysis of PI3K-Akt pathway proteins in MH7A cells treated with HG-9-91-01. (**C–D**) Western blot analysis of NF-κB signalling pathway proteins in MH7A cells treated with HG-9-91-01. (**E–J**) Western blot analysis of Bax, Bcl-2, iNOS, TNF-α and MMP9 expression in MH7A cells treated with HG-9-91-01. All data in the figures are presented as mean±SD of three independent experiments; *p<0.05, ***p<0.001 vs control group. Bax, Bcl-2-associated X protein; Bcl-2, B-cell lymphoma 2; IκBα, inhibitor of nuclear factor kappa B alpha; iNOS, inducible nitric oxide synthase; MMP9, matrix metalloproteinase 9; mTOR, mechanistic target of rapamycin; NF-κB, nuclear factor kappa B; PI3K-Akt, phosphoinositide 3-kinase-protein kinase B; TNF, tumour necrosis factor.

RNA-seq analysis revealed that the effects of HG-9-91-01 on MH7A cells were associated with enrichment in the PI3K-Akt and NF-κB signalling pathways. To further investigate these findings, we examined the expression of key signalling proteins in both pathways using western blot analysis. Results showed that HG-9-91-01 dose-dependently inhibited the activation of the PI3K-Akt pathway, as indicated by reduced phosphorylation levels of PI3K, Akt and mTOR, without changes in their total protein expression (figure 4A,B). Similarly, the compound suppressed the NF-κB pathway, leading to decreased phosphorylation of p65 and inhibitor of nuclear factor kappa B alpha (IκBα), while total protein levels remained unaltered (figure 4C,D).

In line with the inhibition of these pro-survival and inflammatory signalling cascades, HG-9-91-01 also modulated the expression of critical downstream functional proteins. The pro-apoptotic protein Bax was upregulated, while the anti-apoptotic protein Bcl-2, along with pro-inflammatory proteins iNOS and TNF-α, and the invasion-associated protein MMP9, were downregulated (figure 4E,J). Together, these results suggest that HG-9-91-01 exerts anti-inflammatory, pro-apoptotic and anti-invasive actions in association with suppression of the PI3K-Akt and NF-κB signalling axes, leading to coordinated alteration in downstream effector expression.

### HG-9-91-01 alleviates joint pathology and bone damage in CIA mice

**Figure 5:**
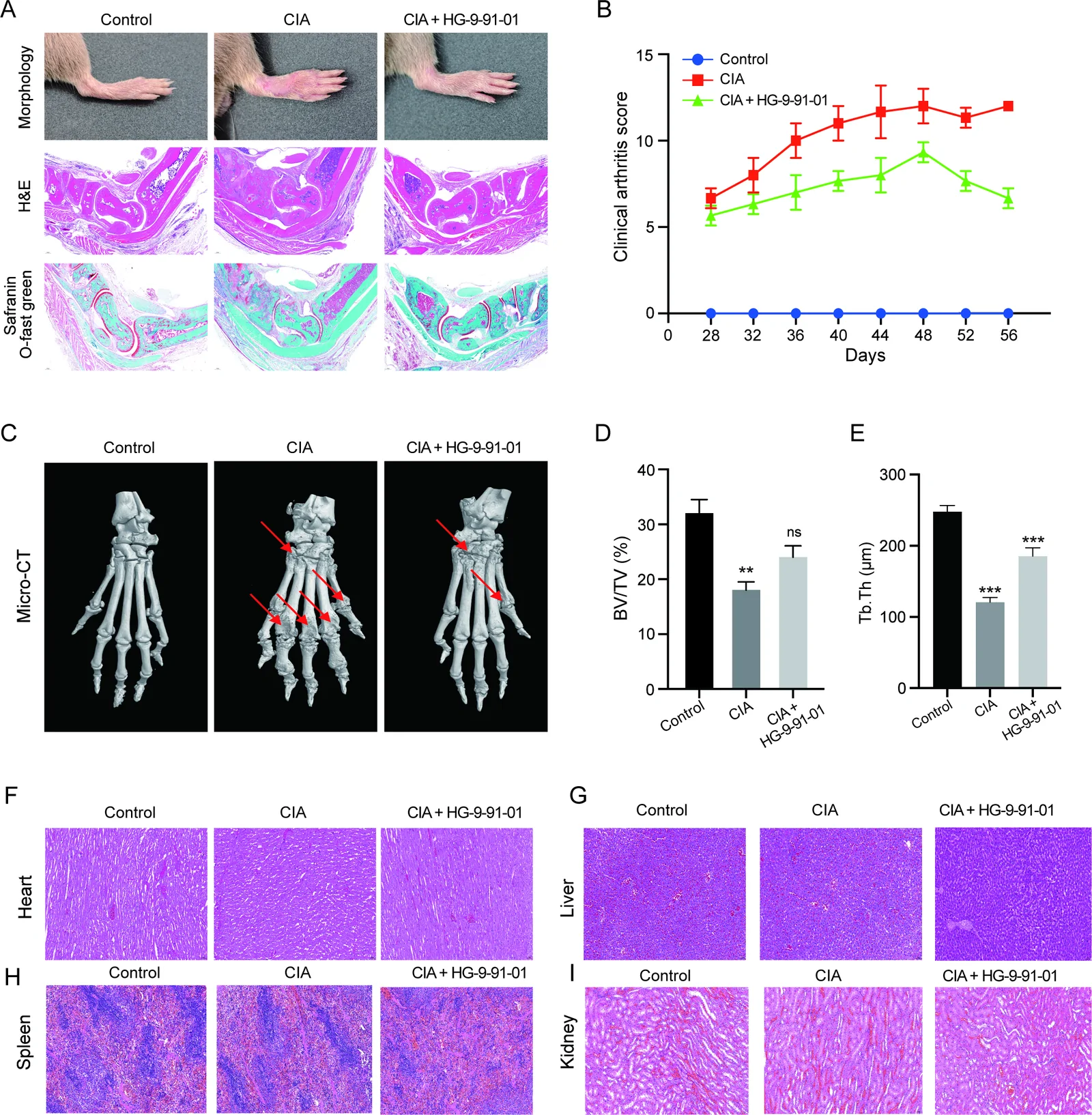
HG-9-91-01 alleviates clinical arthritis severity, joint histopathological damage and bone destruction in CIA mice. (**A**) Representative images of hind paw morphology and joint histological staining (H&E and Safranin O) in control, CIA and CIA+HG-9-91-01 groups. HG-9-91-01 was administered via tail vein injection at a dosage of 10 mg/kg once daily for 28 consecutive days. (**B**) Arthritis scores in control, CIA and CIA+HG-9-91-01 mice. (**C**) Representative micro-CT images of paw bone structure in control, CIA and CIA+HG-9-91-01 mice. (**D**) Quantification of BV/TV. (**E**) Quantification of Tb.Th. (**F–I**) Representative H&E staining images of major visceral organs including heart (**F**), liver (**G**), spleen (**H**) and kidney (**I**). Data are presented as mean±SD (n=6 per group); **p<0.01, ***p<0.001 vs control group. BV/TV, bone volume fraction; CIA, collagen-induced arthritis; ns, not significant; Tb.Th, trabecular thickness.

To assess the therapeutic potential of HG-9-91-01 in RA, we used a CIA mouse model. CIA mice developed robust paw swelling, severe synovial hyperplasia, extensive inflammatory cell infiltration and loss of cartilage matrix. Treatment with HG-9-91-01 effectively alleviated these pathological features and preserved articular cartilage integrity (figure 5A). Consistent with the histological improvement, the clinical arthritis score was also significantly suppressed (figure 5B). Micro-CT imaging and subsequent quantitative analysis further confirmed the protective effect of HG-9-91-01 on bone structures. Representative images revealed extensive bone erosion (red arrows) in the joints of CIA mice, which was substantially attenuated by HG-9-91-01 treatment (figure 5C). Moreover, quantitative analysis of the bone microstructure demonstrated that HG-9-91-01 prevented the reduction in BV/TV and Tb.Th (figure 5D,E).

To assess the potential systemic toxicity of HG-9-91-01, we performed H&E staining on heart, liver, spleen and kidney tissues from HG-9-91-01-treated CIA mice (figure 5F–I). No obvious pathological damage, such as inflammatory infiltration, cellular necrosis or parenchymal structural abnormality, was observed in these major visceral organs. These histological findings demonstrate that HG-9-91-01 at the applied therapeutic dosage exerted no evident organ toxicity under the current administration regimen.

## Discussion

**Figure 6:**
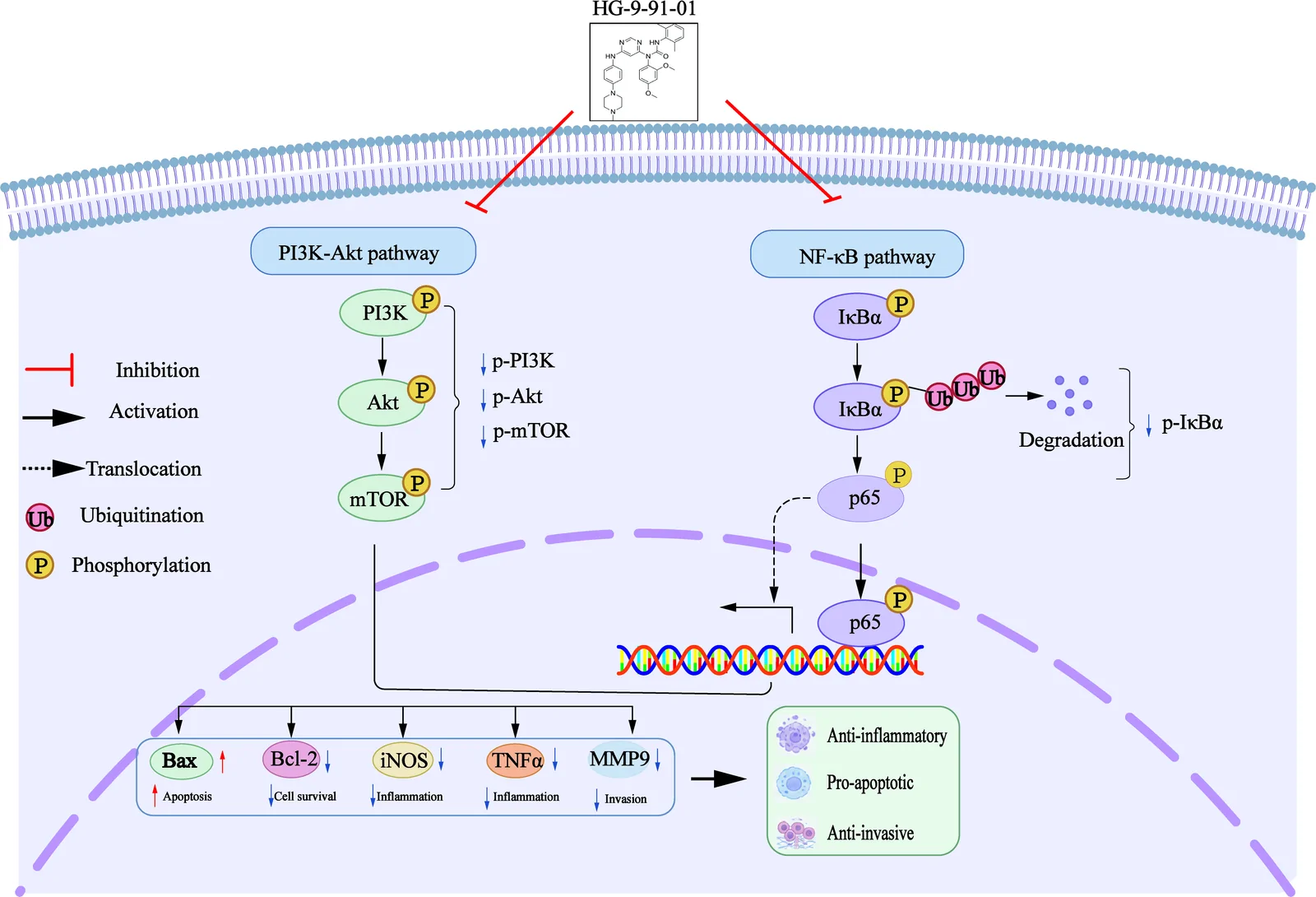
Proposed mechanism of action of HG-9-91-01 in rheumatoid arthritis fibroblast-like synoviocytes. HG-9-91-01 suppresses the activation of the PI3K-Akt and NF-κB signalling pathways. Inhibition of these signalling pathways is associated with increased Bax expression and decreased expression of Bcl-2, iNOS, TNF-α and MMP9, resulting in enhanced apoptosis and reduced inflammatory and invasive phenotypes of MH7A cells. Bax, Bcl-2-associated X protein; Bcl-2, B-cell lymphoma 2; IκBα, inhibitor of nuclear factor kappa B alpha; iNOS, inducible nitric oxide synthase; MMP9, matrix metalloproteinase 9; mTOR, mechanistic target of rapamycin; NF-κB, nuclear factor kappa B; PI3K-Akt, phosphoinositide 3-kinase-protein kinase B; TNF, tumour necrosis factor.

This study demonstrates that pharmacological inhibition of SIKs by HG-9-91-01 effectively attenuates the pathogenic activity of RA-FLS, with these effects associated with suppression of the PI3K-Akt and NF-κB signalling axes (figure 6). Our findings identify SIK3 as a disease-associated isoform in RA synovium and position SIK family inhibition as a promising therapeutic strategy for RA.

The broader context of SIK biology further supports the relevance of targeting this kinase family in RA. The three SIK isoforms have been implicated in diverse physiological and pathological processes: SIK1 regulates blood pressure and tumour metastasis^16 24–27^; SIK2 modulates breast cancer proliferation and adipocyte metabolism^28–31^ and SIK3 drives vascular smooth muscle hyperplasia and ovarian cancer progression.^32–34^ In the context of RA, we found that SIK3 was the predominantly expressed isoform in RA synovium and its expression positively correlated with disease activity. These observations, together with the established role of SIKs in inflammatory signalling, provided the rationale for investigating the therapeutic effects of HG-9-91-01 in RA-FLS.

The mechanistic core of our study lies in the concurrent modulation of two interconnected signalling pathways central to RA-FLS pathogenicity. Specifically, HG-9-91-01 treatment was associated with reduced phosphorylation of PI3K, AKT and mTOR, as well as decreased phosphorylation of p65 and IκBα, indicating suppression of both the PI3K-Akt and NF-κB pathways. This dual pathway inhibition was accompanied by downstream molecular changes—upregulation of Bax, downregulation of Bcl-2 and reduced expression of iNOS, TNF-α and MMP-9—collectively explaining the coordinated suppression of FLS proliferation, migration, invasion and inflammatory activation observed in our functional assays. Given the well-established crosstalk between these signalling cascades in driving FLS activation,^35^ their concurrent inhibition distinguishes HG-9-91-01 from current RA therapies that typically target single pathways and provides a mechanistic rationale for its broad efficacy.

To further explore whether HG-9-91-01 exerts broader effects beyond FLS, we examined its impact on human PBMCs. Treatment with HG-9-91-01 selectively altered immune cell composition in PBMCs from RA patients—increasing the proportions of CD3^+^CD4^+^ T cells and CD4^+^CD25^+^Foxp3^+^ regulatory T cells while reducing CD19^+^ B cells—without affecting immune cell proportions or inducing apoptosis in PBMCs from HCs (online supplemental figure S2). These observations suggest that HG-9-91-01 may also exert certain immunomodulatory effects in the inflammatory milieu of RA, although the underlying mechanisms and functional implications remain to be determined. Importantly, this finding does not diminish the primary conclusion that HG-9-91-01 directly suppresses FLS pathogenicity, but rather raises the possibility of additional immune-mediated contributions to its overall efficacy.

The translational relevance of our findings is strongly supported by the consistent efficacy profile observed across experimental models. In MH7A cells, HG-9-91-01 effectively suppressed the aggressive phenotypes characterising activated RA-FLS, while in the CIA mouse model, it significantly ameliorated both clinical disease parameters and joint histopathology. This concordance between in vitro and in vivo findings underscores the therapeutic potential of SIK family inhibition for RA treatment. Notably, the mechanism of action of HG-9-91-01 addresses a critical limitation of current RA therapies. While conventional DMARDs, biologics and JAK inhibitors primarily target immune activation pathways, they often fail to adequately control the synovial-invasive processes driven by RA-FLS.^36^ Our data suggest that HG-9-91-01 directly targets these fundamental pathological mechanisms, potentially offering superior protection against structural joint damage.

Despite the promising results, several limitations should be acknowledged. All cellular experiments were performed using the MH7A cell line and validation in primary human RA-FLS is essential to confirm the translational relevance of our findings. Additionally, although SIK3 was the predominantly expressed isoform in RA synovium, HG-9-91-01 is a pan-SIK inhibitor, and we cannot definitively attribute its therapeutic effects solely to SIK3 inhibition, as concurrent suppression of other kinases may also contribute to the observed effects. Our investigation of immunomodulatory effects was limited to flow cytometric analysis of cell composition, and we have not assessed the compound’s effects on other joint-resident cells such as chondrocytes and osteoclasts. Furthermore, while 28-day toxicity assessment in mice revealed no obvious organ damage, the long-term safety of pan-SIK inhibition remains uncertain given the ubiquitous functions of SIK isoforms. Future studies addressing these issues—including primary cell validation, isoform-specific genetic experiments, comprehensive immunoprofiling and long-term safety evaluation—will be essential to advance this strategy towards clinical translation.

In conclusion, our work establishes SIK family inhibition as a promising therapeutic strategy for RA and identifies HG-9-91-01 as a compelling candidate for further development. The compound’s ability to concurrently target multiple pathological processes in RA-FLS through coordinated regulation of PI3K-Akt and NF-κB signalling represents a significant advance in the pursuit of more effective RA therapies. Future studies exploring the clinical translation of SIK-targeted approaches, including optimisation of drug-like properties and comprehensive safety profiling, are strongly warranted to realise the full therapeutic potential of this novel strategy.

## Supplementary material

10.1136/rmdopen-2026-007000 online supplemental file 1

10.1136/rmdopen-2026-007000 online supplemental file 2

## Data Availability

Data are available in a public, open access repository. Data are available on reasonable request.
