# Supplementary material for: SIK inhibitor HG-9-91-01 suppresses the pathogenic activity of fibroblast-like synoviocytes in rheumatoid arthritis
Source: RMD Open. 2026 Aug 20;12(3):e007000. doi: 10.1136/rmdopen-2026-007000 (PMC13504899; doi:10.1136/rmdopen-2026-007000)
Supplement: Supplementary file 1 [file rmdopen-12-3-s001.docx]

Supplementary Figure

**Supplementary Figure S1. Single-cell transcriptomic analysis of SIK family expression in synovial immune-cell populations.** (A) UMAP visualization of major cell populations identified from the single-cell RNA-sequencing dataset, including fibroblasts, T cells, B cells, macrophages, endothelial cells, smooth muscle cells, and lymphatic endothelial cells. (B) Violin plots showing the expression levels of SIK1, SIK2, and SIK3 in synovial T cells, B cells, and macrophages from HC and RA samples. Data were analyzed from a publicly available single-cell RNA-sequencing dataset. ns, not significant; **P < 0.01; ***P < 0.001.

**Supplementary Figure S2. HG-9-91-01 selectively modulates immune cell composition in PBMCs from RA patients but not healthy controls.** **PBMCs isolated from HC and patients with RA were treated with HG-9-91-01 (1 μM) or vehicle control for 6 h and analyzed by flow cytometry.** (A–H) Representative flow cytometry plots and quantitative analysis of CD3+CD4+ T cells, CD19+ B cells, CD4+CD25+Foxp3+ regulatory T cells (Tregs), and apoptotic cells in HC-derived PBMCs. HG-9-91-01 did not significantly alter immune cell composition or apoptosis in HC PBMCs. (I–P) Representative flow cytometry plots and quantitative analysis of CD3+CD4+ T cells, CD19+ B cells, CD4+CD25+Foxp3+ Treg cells, and apoptotic cells in RA-derived PBMCs. HG-9-91-01 significantly increased the proportions of CD3+CD4+ T cells and Treg cells, while reducing the proportion of CD19+ B cells, without inducing significant apoptosis. Data are presented as mean ± SD. ns, not significant; **P < 0.01; ***P < 0.001 versus control.
